# Relationship between Lifestyle and Frailty among Iranian Community-Dwelling Older Adults: Pilot Study

**DOI:** 10.14283/jarlife.2023.16

**Published:** 2023-11-28

**Authors:** S. Nazari, M. Bakhtiyary, A.N. Shabestari, F. Sharifi, P.F. Afshar

**Affiliations:** 1 School of Nursing and Midwifery, Tehran University of Medical Sciences, Tehran, Iran; 2 Department of Geriatric Medicine, School of Medicine, Tehran University of Medical Sciences, Tehran, Iran; 3 Elderly Health Research Center, Endocrinology and Metabolism Research Institute, Tehran University of Medical Science, Tehran, Iran; 4 Department of Gerontology, School of Behavioral Sciences and Mental Health (Tehran Institute of Psychiatry), Iran University of Medical Sciences, Tehran, Iran

**Keywords:** Aged, life style, healthy lifestyle, frail elderly, frailty

## Abstract

**Background:**

Aging affects physical, mental, and social functions, which can lead to an increase in frailty. Old adults with frailty syndrome are prone to disabilities and hospitalization. Lifestyle is a context-based factor that has the potential to prevent frailty.

**Objectives:**

This study aimed to assess the relationship between lifestyle and frailty among Iranian community-dwelling older adults.

**Design, Setting:**

This is a descriptive-analytical cross-sectional study. The participants were 513 older adults over 60 years by the convenience sampling method from the retirement center.

**Measurements:**

Data were collected using Tilberg’s frailty index, the Iranian elderly lifestyle questionnaire, and the Mini-Cog test. Data were analyzed with SPSS v.26 software by chi-square and logistic regression tests.

**Results:**

The age of the participants was 66.43 ± 4.69 years. The male-to-female sex ratio was 1.5 (39.2% women). The lifestyle of 96 (19.3%) old adults was unfavorable. 18.7 percent of older adults had Frailty syndrome. The logistic regression test showed that moderate and favorable lifestyle (OR= 0.06; 95% CI: 0.02-0.16), age over 75 years (OR= 5.25; 95% CI: 2.35-11.69), retired employment status (OR= 0.13; 95% CI: 0.29-0.05) are factors that have a significant relationship with frailty (P< 0.05).

**Conclusion:**

The findings showed that lifestyle can predict frailty. Therefore, it seems that an optimal lifestyle can prevent the frailty of older adults.

## Introduction

**A**ge-related changes adversely affect normal functions such as physical, psychological, and social functioning (1-3). Frailty syndrome is a set of defects that ultimately causes a decrease in physiological reserve capacities and fragility against stressful factors (4). The incidence of frailty varies among older adults. The prevalence of frailty syndrome varies between 0.4% and 59.1%, depending on the criteria. The prevalence of frailty in low and middle-income countries is around 18%, in high-income countries is 10% (5-8).

The prevalence of CI in Nigeria is less studied than in high income countries . In a survey of cognitive impairment among Yoruba speaking sample from Ibadan Nigeria, 152 (62%) out of 423 individuals studied were diagnosed with cognitive impairment no dementia (CIND) while 28 (6.61%) were diagnosed with dementia (7). In northern Nigeria (8), survey of 323 older adults showed dementia prevalence at 2.79% (CI 1– 4.58%) representing 66.67% of all the cases of dementia in the sample. In south-west Nigeria, 10.1% prevalence of probable dementia were found (9) using the 10 Word Delay Recall test adapted from Consortium to Establish a Registry for Alzheimer’s Disease CERAD (10) . In the North Central Nigeria, Ochayi and Thatcher (11) using the Community Screening Instrument for Dementia (CSID), showed a 6.4% overall prevalence of dementia and in south east Nigeria, 23.1% depression prevalence was shown in older adult sample with 20.7% complaining of forgetfulness (12).

Old adults with frailty syndrome are more vulnerable to health-related problems, including falls, delirium, fractures, disabilities, hospitalizations, and death (9-11). Frailty is associated with energy imbalance, sarcopenia, and reduced function and strengh (12). Some studies have shown that several risk factors can increase the incidence of frailty syndrome, including demographic characteristics (such as old age, female, low educational status, and unfavorable economic status), multiple chronic diseases, malnutrition, and insufficient physical activity, cognitive disorders, and poor function (13-16). Some of these factors are in the lifestyle field. Successful aging is the opposite of frailty, and a healthy lifestyle can predict successful aging (17). Lifestyle is related to the dimensions of nutrition, physical activity, sleep and daily patterns, so it is possible to improve the organs reserve and prevent vulnerability (18).

A person’s lifestyle includes physical, mental, and social domains (19, 20). World Health Organization (WHO) stated lifestyle is approximately 60% of the quality of life related to health (19). Lifestyle is defined in two levels macro (society) and micro (individual-level). The micro level refers to diet and physical activity, alcohol use, smoking, habits, choices, goals, and beliefs (21). The macro level refers to consumption behaviors, social support, social cohesion. People choose their own lifestyle and generally people’s behavior is the result of their choices in the available opportunities (22). The lifestyle is very culture-oriented and varies according to different societies. An unhealthy lifestyle is associated with an increase in mortality (23). It has been stated that a healthy lifestyle can reduce the death rate from chronic diseases by 50% (24).

Lifestyle is influenced by culture and environmental conditions (23, 25). On the other hand, a healthy lifestyle can predict successful aging. Therefore, it can be assumed that frailty maybe is influenced by lifestyle, and it is necessary to examine lifestyle in a context-based method. Lifestyle is a behavioral and situational framework in every person’s life. But first, it is necessary to assess these questions: Is lifestyle related to frailty? Can lifestyle affect frailty? This study cannot answer a comprehensive response to these questions, but it can be a start for future studies. So, this study aimed to assess the relationship between lifestyle and frailty among Iranian community-dwelling older adults.

## Method

### Design Study

This is a descriptive-analytical cross-sectional study. This is a pilot study. The research population was elderly people aged 60 and above from the retirement center of the Tehran University of Medical Sciences.

### Sampling Method

The sampling method was convenient in this study. The sample size was calculated using the formula n= . 
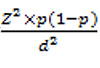
The prevalence of frailty is about 14.3% (26, 27). Z= 1.96 and d is considered to be 0.3. The sample size was 523 people. Five questionnaires were incomplete and five people were excluded from the study due to cognitive impairment. The sample size was 513 people.

### Inclusion and exclusion criteria

The inclusion criteria included the willingness to participate in the study, and the ability to communicate, and the exclusion criteria included movement limitations, hearing and vision impairments, and cognitive disorders (according to Mini-Cog), incomplete questionnaire.

### Measurements

#### Demographic characteristics

Demographic characteristics include age, sex, education, employment status (employed, retired, unemployed), the number of co-morbidities.

#### Tilburg Frailty Indicator (TFI)

Gobbens et al. developed TFI in 2010. TFI consists of two parts. Part A contains ten questions including age, sex, education and income, marital status, country of birth, types of Stressful Life Events in the past year, comorbidities, place satisfaction, and self-evaluation of living conditions. Part B refers to the main factors of frailty and includes fifteen questions that are divided into three physical, psychological, and social dimensions. Eleven questions are answered with two options (yes and no) and four questions with three options (yes, no, and sometimes). The physical dimension includes eight questions about physical health (physical function), unwanted weight loss, difficulty walking, difficulty maintaining balance, hearing impairment, visual impairment, reduction (lack of) strength in hands, and physical fatigue. The psychological dimension includes four questions related to cognitive status, depression, neurological symptoms, as well as coping with problems, and finally, the social dimension also includes three questions related to living alone, social relationships, and social support (28). The scoring of TFI is from zero to fifteen and the cut point is five. A score of five or more is considered to mean an elderly person is frail. Cronbach’s alpha was 0.81 in the Persian version of TFI and its validity has confirmed the existence of all three dimensions using the construct validity method. The accuracy of this index was 0.88. Its sensitivity and specificity in the point 4.5 cut-offs were obtained as 0.95 and 0.86 in a study by Mazzuchi et al. (2020) (29). Cronbach’s alpha was 0.71 in this study.

#### The Healthy lifestyle assessment questionnaire

The Healthy lifestyle assessment questionnaire was designed by Eshaghi et al. in 2007. This questionnaire contains 46 questions, which include fifteen questions about prevention, fourteen questions about healthy nutrition, five questions about stress management, seven questions about social and interpersonal relationships, and five questions about physical activity, exercise, recreation, and entertainment. The face and content validity has been confirmed and its Cronbach’s alpha was 0.76. The scoring of this questionnaire is done in the form of a Likert scale from one to five. The lowest score of the questionnaire is 42 and the highest score is 211. The total score is placed in one of three levels « undesirable, medium, and optimal». Score 42-98: undesirable lifestyle, score 99-155: medium lifestyle, and score 156-211: optimal lifestyle. This tool could be used in the Iranian elderly population due to its simplicity of sentences, as well as appropriate validity and reliability (30). Cronbach’s alpha was 0.97 in this study.

#### Mini-Cog test

The Mini-Cog test is a screening test used to identify people with cognitive disorders (31). The evaluation time is about three to five minutes (32). The older adult is taught to memorize three unrelated words together, and we ask him to repeat those three words. Then the clock-drawing test is assessed by drawing the clock. After that, we asked older adults those three words again. To calculate the score of this test, we will give one point for each correctly remembered word out of the three. If the older adults cannot remember the three words, they may have a cognitive disorder category (score = zero). Still, if they remember all three words correctly, they will be in the non-cognitive disorder category (score = 3). Older adults who only remember one or two words are divided into two groups based on the results of the clock drawing test: if the clock test is correct, the older adult is considered to have no cognitive impairment, but if his/ her clock test was also impaired, then it means that he has a cognitive disorder (33). Rezaei et al. psychometrically evaluated Mini-Cog in Iranian older adults. Cronbach’s alpha was 0.83. Its sensitivity and specificity were 88% and 63%, respectively (34).

### Ethical considerations

We confirm that this study was following the guidelines and regulations of the Declaration of Helsinki. This study was approved by the research ethics committee of the Tehran University of Medical Sciences (ref.: IR.TUMS.MEDICINE.REC.1400.638). We explained the objectives to the participants and obtained informed written consent.

### Data analysis

Descriptive statistics were shown by frequency, mean, and standard deviation. Data were analyzed using chi-square tests and logistic regression. The normality of the data was also determined using the Kolmogorov-Smirnov test. Data were analyzed using SPSS v.26.

## Results

The age of the participants was 66.43 ± 4.69 years. The participants included 201 (39.2%) women and 312 (60.8%) men. 96 old people (18.7%) have Frailty syndrome, and 99 people (19.3%) have an unfavorable lifestyle (other information is shown in Table 1).

**Table 1.** Demographic characteristics of study participants

	Variable	F (%)
Marriage	Married	324 (63.2)
	Single	44 (8.6)
	Divorced	18 (3.5)
	Widow	127 (24.8)
	Total	513 (100)
Education	Illiterate	28 (5.5)
	Reading & writing	93 (18.1)
	Middle school	104 (20.3)
	High school	147 (28.7)
	Academic degree	141 (28.7)
	Total	513 (100)
Comorbidity	Yes	341 (66.5)
	No	172 (33.5)
	Total	513 (100)
Employment status	employed	109 (21.2)
	retired	383 (74.7)
	unemployed	21 (4.1)
	Total	513 (100)
Frailty	Nom-Frail	417 (81.3)
	Frail	96 (18.7)
	Total	513 (100)
Lifestyle	Undesirable	99 (19.3)
	medium	200 (39)
	Optimal	214 (41.7)
	Total	513 (100)

The average scores of frailty and lifestyle of the elderly in this study were 3.69 ± 2.579 and 146.15 ± 40.174, respectively. The Mean and standard deviation of their dimensions are shown in Table 2. The distribution was non-normal in all frailty and lifestyle subscales based on the Kolmogorov-Smirnov test (P< 0.01).

**Table 2.** Mean and standard deviation of frailty and lifestyle dimensions

		Mean ± SD	Min	Max
Frailty	Physical dimension	1.72 ± 0.16	1.13	2.00
	Psychological dimension	1.97 ± 0.26	1.25	2.75
	Social dimension	1.73 ± 0.24	1.00	2.33
Lifestyle	Prevention	16.88 ± 4.19	7.33	23.67
	Healthy nutrition	15.14 ± 4.71	6.07	23.57
	Stress management	13.92 ± 4.48	6.00	24.00
	Social relationships	16.03 ± 5.21	7.14	24.29
	Physical activity	16.52 ± 4.77	5.00	25.00

The highest frailty was seen in over 75 years of age (30.1%), women (18.9%), single (61.4%), and illiterate (46.4%) (Table 3).

**Table 3.** Frequency of frailty based on demographic characteristics

Variable	Frail (%)	Non-Frail (%)	Total	Contingency Coefficient	P
Age				0.179	<0.001
60-74 yrs.	25 (11.21)	198 (88.79)	223 (43.5)		
75-84	46 (22.22)	161 (77.78)	207 (40.4)		
>85	25 (30.12)	58 (69/88)	83 (16.1)		
Sex				0.004	0.929
Male	58 (18.6)	254 (81.4)	312 (60.81)		
Female	38 (19.9)	163 (80.1)	201 (39.19)		
Marriage				0.240	<0.001
Married	33 (10.2)	291 (89.8)	324 (63.16)		
Single	27 (61.4)	17 (38.6)	44 (8.57)		
Divorced	7 (38.9)	11 (61.1)	18 (3.51)		
Widow	31 (24.4)	96 (75.6)	127 (24.76)		
Education				-0.231	<0.001
Illiterate	13 (46.6)	15 (53.6)	28 (5.46)		
Reading & writing	27 (29)	66 (71)	93(18.13)		
Middle school	28 (26.9)	76 (73.1)	104 (20.27)		
High school	13 (8.8)	134 (91.2)	147 (28.65)		
Academic degree	17 (12.1)	124 (87.9)	141 (27.49)		
Comorbidity				-0.104	0.019
Yes	75 (22)	266 (78)	341 (66.47)		
No	23 (13.4)	149 (86.6)	172 (33.53)		
Employment status				-0.107	0.015
Employed	29 (26.6)	80 (73.4)	109 (21.25)		
Retired	67 (17.5)	316 (82.5)	383 (74.66)		
Unemployed	2 (9.5)	19 ()	21 (4.09)		
Lifestyle				-0.382	<0.001
Undesirable	58 (58.6)	41 (41.4)	99 (19.30)		
medium	22 (11)	178 (89)	200 (38.98)		
Optimal	18 (8.4)	196 (91.6)	214 (41.72)		

The results of the logistic regression showed that lifestyle, age, employment status are factors that have a significant relationship with frailty (Table 4). Above 75 years of age is a risk factor for frailty (OR= 5.25; 95% CI: 2.35-11.69). A medium and optimal lifestyle (OR= 0.06; 95% CI: 0.02-0.16), retired employment status (OR= 0.13; 95% CI: 0.05-0.29) were protective factors. The result of the Hosmer and Lemeshow Test was (P= 0.35).

**Table 4.** Logistic regression of frailty and related factors

Variables	Logistic regression		
	Exp(B)	95% confidence interval	P
Lifestyle (Undesirable)			
medium	0.06	(0.02-0.15)	<0.001
Optimal	0.06	(0.02-0.16)	<0.001
Sex (male)	2.24	(1.11-4.56)	0.06
Age (60-74 yrs.)			
75-84	5.25	(2.35-11.69)	<0.001
>85	5.27	(1.82-15.23)	0.002
Marriage (Married)			
Single	2.56	(0.77-8.54)	0.126
Divorced	1.13	(0.24-5.30)	0.873
Widow	1.31	(0.43-2.18)	0.945
Education (Illiterate)			
Reading & writing	0.67	(0.18-2.42)	0.544
Middle school	0.85	(0.23-3.15)	0.804
High school	0.22	(0.05-0.94)	0.051
Academic degree	0.37	(0.08-1.75)	0.210
Employment status (Employed)			
Retired	0.13	(0.05-0.29)	<0.001
Unemployed	0.01	(0.001-0.07)	<0.001
Number of comorbidities (Zero)			
One	0.97	(0.20-4.60)	0.969
Two	1.13	(0.19-6.49)	0.890
Three	0.30	(0.04-2.27)	0.245
Four	0.23	(0.01-3.66)	0.299
Constant	7.38	-	0.050

## Discussion

This study showed that 18.7% of the old participants had frailty. The findings showed that there is a significant relationship between frailty syndrome and lifestyle. An optimal lifestyle is associated with a decrease in the frailty of old people.

The prevalence of frailty in other studies was estimated as 14.3% to 33.4% (35, 36). A study found that the prevalence of frailty was about 24% among community-dwelling older adults (37). Many reasons can explain these differences in the studies. The first reason is the different frailty measurement tools because each of these tools can measure various components of frailty and even focus on a series of specific dimensions of frailty. Also, this difference could be the sampling method. The second reason is the statistical population; if nursing homes or hospitals are selected for sampling, we will likely see a higher prevalence of frailty.

Participants who had an optimal lifestyle were less likely to suffer from frailty syndrome, optimal lifestyle can be one of the protective factors to prevent this syndrome. For example, an old person who does not comply with risk prevention and personal hygiene or does not have a proper diet, or does not have enough daily physical activity, has a high chance of suffering from frailty. On the contrary, those who have an optimal lifestyle, that is, follow health and preventive measures well, have proper nutrition and physical activity, and have good psychological and social conditions, are less likely to get frailty syndrome. Gobens et al. concluded that lifestyle cannot predict frailty (38). the results of the research by Khodamoradi et al. show the existence of modifiable risk factors such as obesity and insufficient physical activity, which are important. It is necessary to use appropriate strategies to prevent frailty, due to the complications and high costs of frailty.

Katayama et al. found that elderly with physical frailty have reduced any activity in their lifestyle including social activities, physical and cognitive activities. Older adults with frailty showed a significant relationship with fewer activity patterns compared to non-frail elderly. Katayama et al stated that frail elderly suffer from disturbances in activity patterns (19).

Abe et al found that it was seen with a lower probability of frailty and its related consequences in participants who did agriculture, sports, activity, and social participation (39). The results of Wang et al.’s study also indicated that participation in social activities was less among people who were frail than non-frail old people. In addition, frailty risk decreased with a healthy diet in old age (40).

This study showed that there is a significant relationship between age and frailty. People with frailty in this study are generally in the age range of 60 to 75 years. The highest prevalence of frailty is seen in people over 75 years old. We can conclude that physical and mental capacities decrease with aging and the possibility of suffering from frailty syndrome increases (41-43).

This study showed that there is no significant difference in frailty between women and men. Some studies have stated that the prevalence of frailty is higher in women (5, 44, 45), and some studies found that frailty is higher in men than women (43, 46). On the other hand, some studies showed that there is no significant relationship between gender and frailty (41, 47, 48). Demographic and community differences can partially explain these variable results.

The frequency of frail old people in retirees was higher than in other employment statuses, but this is due to the larger number of people in this category. The highest percentage of frailty is in the employed category. Employed elderly probably have jobs that are not suitable for their physical and mental conditions due to their financial needs. Unsuitable working conditions can put the elderly under all kinds of physical and mental pressures, and as a result, put them in conditions where they are prone to or suffer from frailty syndrome. Previous studies had found that there is a significant relationship between employment status and frailty, they found that the employed elderly have the least frailty, and this disparity could be due to the difference in people’s jobs or volunteer activities (26, 49).

## Conclusion

According to the results of this study, the prevalence of frailty was 18.7%. Lifestyle is related to all physical, mental, and social aspects of people. The state of frailty, especially in the elderly, is directly related to lifestyle. Probably, frailty is reduced by improving lifestyle.

### Limitations

This study coincided with the covid-19 epidemic, which led to reduced cooperation of participants, which may have affected the data and results. This is a pilot study and it is necessary to conduct it in the future in a larger and more diverse population. It would have been better to separate the lifestyle dimensions, but the Healthy lifestyle assessment questionnaire did not have this possibility.
